# Circuit-Specific Control of Blood Pressure by PNMT-Expressing Nucleus Tractus Solitarii Neurons

**DOI:** 10.1007/s12264-022-01008-3

**Published:** 2023-01-02

**Authors:** Shirui Jun, Xianhong Ou, Luo Shi, Hongxiao Yu, Tianjiao Deng, Jinting Chen, Xiaojun Nie, Yinchao Hao, Yishuo Shi, Wei Liu, Yanming Tian, Sheng Wang, Fang Yuan

**Affiliations:** 1 Department of Neurobiology, Hebei Medical University, Shijiazhuang, 050017 China; 2 Institute of Cardiovascular Research, Southwest Medical University, Luzhou, 646000 China; 3 Core Facilities and Centers, Institute of Medicine and Health, Hebei Medical University, Shijiazhuang, 050017 China; 4 Hebei Key Laboratory of Neurophysiology, Shijiazhuang, 050017 China

**Keywords:** Nucleus tractus solitarii, Blood pressure, Rostral ventrolateral medulla, Optogenetics, Neural circuit

## Abstract

The nucleus tractus solitarii (NTS) is one of the morphologically and functionally defined centers that engage in the autonomic regulation of cardiovascular activity. Phenotypically-characterized NTS neurons have been implicated in the differential regulation of blood pressure (BP). Here, we investigated whether phenylethanolamine N-methyltransferase (PNMT)-expressing NTS (NTS^PNMT^) neurons contribute to the control of BP. We demonstrate that photostimulation of NTS^PNMT^ neurons has variable effects on BP. A depressor response was produced during optogenetic stimulation of NTS^PNMT^ neurons projecting to the paraventricular nucleus of the hypothalamus, lateral parabrachial nucleus, and caudal ventrolateral medulla. Conversely, photostimulation of NTS^PNMT^ neurons projecting to the rostral ventrolateral medulla produced a robust pressor response and bradycardia. In addition, genetic ablation of both NTS^PNMT^ neurons and those projecting to the rostral ventrolateral medulla impaired the arterial baroreflex. Overall, we revealed the neuronal phenotype- and circuit-specific mechanisms underlying the contribution of NTS^PNMT^ neurons to the regulation of BP.

## Introduction

Neural control of cardiovascular homeostasis plays a critical role in maintaining a sufficient O_2_ supply in mammals. A regulatory hierarchy encompasses complicated, interconnected, and interactive circuits located in the spinal cord, brainstem, hypothalamus, limbic system, and cortex [1]. The arterial baroreflex, one of the crucial mechanisms responsible for the neural control of blood pressure (BP), recruits medullary neural circuits that are at least formed by baroreceptors, the petrosal and nodose ganglia, glutamatergic neurons in the nucleus tractus solitarii (NTS), GABAergic neurons in the caudal ventrolateral medulla (CVLM) and catecholaminergic neurons in the rostral ventrolateral medulla (RVLM) [2]. Neural circuits in the hypothalamus, limbic system, and cortex, together with medullary circuits, contribute to the stress-related, behavioral, and emotional regulation of cardiovascular function [3].

The NTS, receiving visceral afferents and relaying information to numerous autonomic centers, plays a pivotal role in the autonomic regulation of cardiovascular, respiratory, and gastrointestinal activities [1]. The neural control of cardiorespiratory activity has been extensively studied in different subgroups of NTS neurons, including glutamatergic [4–6], GABAergic [7, 8], and catecholaminergic neurons [9, 10]. Of those neurotransmitter-based subgroups, catecholaminergic neurons are divided into dopaminergic, noradrenergic, and adrenergic. These neurons are solely or double-labeled by tyrosine hydroxylase (TH), dopamine beta-hydroxylase (DβH), and phenylethanolamine N-methyltransferase (PNMT). The largest cluster of PNMT-immunoreactive neurons (termed C1) is distributed in the RVLM, the physiological role of which has been comprehensively studied. The only other PNMT-expressing neurons (termed C2/C3) are distributed in the NTS and the rostral part of the dorsomedial medulla [11]. Recently, state-of-the-art genetic and neurobiological approaches have boosted studies of the circuit- and cell-type-specific control of cardiovascular activity. For example, catecholaminergic NTS neurons have been implicated in the regulation of anorexia [12] and blood glucose [13], as well as arterial pressure [14]. Although the cardiovascular role of catecholaminergic neurons has been physiologically and pharmacologically studied, it remains unknown whether those effects are achieved by recruiting neuronal phenotype- and circuit-specific mechanisms. In particular, the cardiovascular contribution of PNMT-expressing NTS neurons (NTS^PNMT^), putative C2 neurons, needs to be elucidated.

Here, we used an optogenetic approach to selectively stimulate NTS^PNMT^ neurons in a PNMT-Cre mouse line to examine cardiovascular effects. Moreover, we further tested whether NTS^PNMT^ neurons are responsible for the arterial baroreflex by performing genetic ablation-based loss-of-function experiments. We found that NTS^PNMT^ neurons not only contributed to the regulation of BP in neuronal phenotype- and circuit-specific mechanisms but also contributed to regulation of the arterial baroreflex.

## Materials and Methods

### Ethics Approval

PNMT-Cre mice of either sex (10–15 weeks old) used in this study were kindly provided by Dr. Ming Lei from the University of Oxford and have been validated [15, 16]. R26-ChR2-tdTomato mice (Strain# 012567) were purchased from the Jackson Laboratory (USA). PNMT-Cre-ChR2-tdTomato mice were generated by crossing PNMT-Cre mice with R26-ChR2-tdTomato mice. All the mice were housed under program-controlled temperature (21 ± 1°C) and humidity (50% ± 10%) with a fixed 12-h light/12-h dark cycle and with *ad libitum* access to food and water. All experiments were performed in accordance with the Guide for the Care and Use of Laboratory Animals and were approved by the Animal Care and Ethics Committee of Hebei Medical University (#Hebmu-2019001). At the conclusion of the experiments, the mice were killed with an overdose of sodium pentobarbital (>100 mg/kg, i.p.), and subsequently decapitated to assure death.

### Stereotaxic Surgery

We used the following viral vectors: AAV2/8-hSyn-DIO-EGFP (OBiO Technology Co., Ltd., China), AAV2/8-CAG-DIO-taCasp3-TEVp (Taitool Bioscience Co., Ltd, China), AAV2/9-EF1α-fDIO-taCasp3-TEVp-WPRE-pA (Taitool Bioscience Co., Ltd), AAV9-EF1α-DIO-ChR2-GFP (Genechem Co., Ltd, China), AAV_retro_-EF1α-DIO-ChR2-GFP (Genechem Co., Ltd), and AAV_retro_-Flex^LoxP^-Flpo (Genechem Co., Ltd). All virus titers were >10^12^ GC/mL and were stored in aliquots at −80°C until use.

All viral injections were made in a motorized stereotaxic frame (RWD, China) based on previously described protocols [17]. In short, mice were anesthetized with pentobarbital sodium (60 μg/g, i.p.). Additional anesthetic was administered as necessary (30% of the original dose). The depth of anesthesia was assessed every 30 min by the absence of corneal and hind-paw withdrawal reflexes. All surgical procedures were carried out under strict aseptic conditions. Each mouse was placed in a prone position on a stereotaxic device (RWD, China). The body temperature was maintained at 37°C by a program-controlled heating pad. Each injection was made using a virus-filled glass pipette (~25 μm tip diameter) connected to a syringe pump (Harvard Apparatus, USA). The pipette was left in place for at least 5 min before withdrawal. After that, each mouse received injections of antibiotic ampicillin (125 mg/kg, i.p.) and the analgesic ketorolac (4 mg/kg, i.p.). Mice were then allowed four weeks to recover before the next experimental measurements.

AAV2/8-hSyn-DIO-EGFP, AAV9-EF1α-DIO-ChR2-GFP, AAV2/8-CAG-DIO-taCasp3-TEVp, or AAV2/9-EF1α-fDIO-taCasp3-TEVp-WPRE-pA were bilaterally injected into the NTS (80 nL per injection, 6 injections) at the level of the calamus scriptorius [AP (anterior/posterior) 0.1 mm, ML (medial/lateral) ± 0.2 mm, DV (dorsal/ventral) − 0.1 mm; AP 0.3 mm, ML ± 0.2 mm, DV − 0.1mm; AP 0.5 mm, ML ± 0.2 mm, DV − 0.1 mm). AAV_retro_-EF1α-DIO-ChR2-GFP, AAV_retro_-EF1α-DIO-mCherry, and AAV_retro_-Flex^LoxP^-Flpo were bilaterally injected into the RVLM (bregma: AP −6.50 mm to −6.65 mm; ML ± 1.28 mm; DV −6.50 mm). All the stereotaxic coordinates refer to The Mouse Brain in Stereotaxic Coordinates [18].

### Physiological Recording

To monitor BP and heart rate (HR) in anesthetized mice, they were injected (i.p.) with urethane (800 mg/kg) plus α-chloralose (40 mg/kg). The depth of anesthesia was evaluated by the absence of corneal and hind-paw withdrawal reflexes. All surgical procedures were carried out under strict aseptic conditions. A cannula was inserted into the jugular vein for drug infusion and a tracheostomy was performed. The left common carotid artery was intubated with a cannula (PE50) filled with heparin-saline. Before use, the PE50 tubing was warmed and stretched to a thin cannula exactly fitting the diameter of the carotid artery. The cannula was connected to a pressure transducer and signals were input into the PowerLab System (AD Instruments, Canada) to record BP and HR.

Telemetric recordings of BP and HR were made in conscious mice as previously described [19]. In short, mice were anesthetized with isoflurane (2%–3%) after initial exposure to a chamber filled with isoflurane for 1–2 min. The depth of anesthesia was assessed by the absence of corneal and hind-paw withdrawal reflexes. All surgical procedures were conducted under strict aseptic conditions. The pressure catheter was inserted into the right common carotid artery and the body of its transmitter was placed subcutaneously in the abdominal area. The transmitters (HD-X11) sent the physical signals (BP and HR) to a receiver and a data-exchange matrix. After wound closure, the mice received injections of the antibiotic ampicillin (125 mg/kg, i.p.) and the analgesic ketorolac (4 mg/kg, i.p.). At least 5 days after recovery, systolic blood pressure (SBP), diastolic blood pressure (DBP), and HR were measured at a sampling rate of 1 kHz. Data were collected and analyzed by a telemetry system (Ponemah v6.00, Data Sciences International, USA).

### Optogenetics

To selectively stimulate NTS^PNMT^ neurons, an optogenetic approach was applied as previously described [17]. Viral vectors incorporating Channelrhodopsin-2 (ChR2) were bilaterally injected into either the NTS (AAV9-EF1α-DIO-ChR2-GFP) or the RVLM (AAV_retro_-EF1α-DIO-ChR2-EGFP) in PNMT-Cre mice. For blue light stimulation of ChR2-expressing neurons, optical fibers (diameter: 200 μm) were connected to a 473-nm LED source (Newdoon Inc., China) that generated 10-ms light pulses at 20 Hz. The output power at the end of the optical fiber was 8–10 mW in all experiments, as measured with an optical power meter (PM20; Thorlabs, USA). In the control experiments, mice were injected with a virus (AAV9-EF1α-DIO-GFP) that was devoid of the ChR2 gene, followed by optogenetic stimulation using the same protocol.

To illuminate the NTS in anesthetized mice, an occipital craniotomy was made to expose the dorsal surface of the medulla oblongata over the NTS. ChR2-transduced NTS neurons were photostimulated by placing an optical fiber over the NTS on one side. After a small hole was drilled in the skull at a given location, optical fibers were placed over the following regions on one side to illuminate the axon terminals projected by NTS^PNMT^ neurons: the paraventricular nucleus of the hypothalamus (PVN; AP −0.44 mm; ML ±0.15 mm; DV −4.90 mm), the lateral parabrachial nucleus (LPBN; AP −5.00 mm to −5.20 mm; ML ±1.45 mm; DV −3.40 mm), the caudal ventrolateral medulla (CVLM; AP −7.20 mm to −7.35 mm; ML ±1.30 mm; DV −6.50 mm), and the RVLM (AP −6.50 mm to −6.65 mm; ML ±1.28 mm; DV −6.50 mm).

To illuminate the NTS in conscious mice, a small hole was drilled in the occiput in anesthetized mice (pentobarbital sodium, 60 μg/g, i.p.) 3 weeks after injection of the virus. A fiber optic cannula was inserted and placed over the NTS on one side. A glue composite was then used to fix the cannula in place, and the skin was closed with a suture. Following a 1-week period of recovery from surgery, the mice were used for optogenetic stimulation. Before optogenetic stimulation with blue light, the implanted fiber optic cannula was attached *via* a ceramic mating sleeve to a multimode fiber-optic rotary joint patch cable, allowing freedom of movement of the mice.

### Ablation of NTS^PNMT^ Neurons

To genetically ablate NTS^PNMT^ neurons, a virus incorporating the Casp3 gene (AAV2/8-CAG-DIO-taCasp3-TEVp) was injected bilaterally into the NTS in PNMT-Cre-tdTomato mice as previously reported [17, 20]. Immunohistochemical assays were carried out to validate ablation efficiency. The tdTomato-expressing neurons were counted in 10 coronal sections from each mouse (bregma, − 7.9 mm to − 7.0 mm; thickness, 25 μm; each separated by 75 μm). To ablate NTS^PNMT^ neurons projecting to the RVLM, the virus incorporating the Casp3 gene (AAV2/9-EF1α-fDIO-Casp3) and the virus encoding GFP (AAV2/8-hSyn-DIO-GFP) were mixed and injected into the NTS of PNMT-Cre mice, while a retrograde virus (AAV_retro_-Flex^LoxP^-Flop) was injected into the RVLM. Then, the neurons expressing GFP were counted in 10 coronal sections from each mouse, as described above. In control experiments, a virus devoid of the Casp3 gene was injected for immunohistochemical and functional experiments.

### Analysis of Baroreflex Function

The arterial baroreflex was assessed based on responses of HR to pressor and depressor effects in anesthetized mice (urethane at 800 mg/kg and α-chloralose at 40 mg/kg) as previously reported [19]. In short, a cannula filled with saline was inserted into the jugular vein for the administration of drugs, while a cannula filled with heparin saline was inserted in the carotid artery to record BP and HR diurnally at a sample rate of 1 kHz using a pressure transducer connected to the PowerLab System (AD Instruments, Canada). To evoke pressor and depressor responses, phenylephrine (PE, 10, 20, and 40 μg/kg) and sodium nitroprusside (SNP, 30, 60, and 120 μg/kg) were intravenously administered in random order. For each response, the change in HR at the time of peak SBP was chosen and ΔHR/ΔSBP was used as an index of baroreflex gain.

### Single-cell Reverse Transcriptase-PCR

Single-cell reverse transcriptase (RT)-PCR (scPCR) was applied to dissociated neurons from brainstem slices prepared from adult PNMT-Cre mice (either sex) with an injection of AAV-hSyn-DIO-EGFP, essentially as described previously [21, 22]. Mice were anesthetized with urethane (1.8 g/kg, i.p) and rapidly decapitated; the brainstem was dissected out and sliced in a coronal plane in ice-cold sucrose-substituted Ringer’s solution containing the following (in mmol/L): 260 sucrose, 3 KCl, 5 MgCl_2_, 1 CaCl_2_, 1.25 NaH_2_PO_4_, 26 NaHCO_3_, 10 glucose, and 1 kynurenic acid, bubbled with 95% O_2_ and 5% CO_2_. The slices were incubated for 15 min at room temperature in PIPES [piperazine-N,N′-bis(2-ethanesulfonic acid)] buffer (in mmol/L): 120 NaCl, 5 KCl, 1 CaCl_2_, 1 MgCl_2_, 25 D-glucose, 20 PIPES, 100% O_2_ and then for 60 min at 33°C in PIPES buffer containing trypsin (Sigma-Aldrich, type XI; 0.5 mg/mL). After enzymatic treatment, the slices were rinsed and maintained in PIPES buffer at room temperature for 60 min. The slices were transferred to DMEM buffer (Invitrogen, Life Technologies) and the NTS was excised under a fluorescence-equipped dissecting microscope (Zeiss Stemi 305). This tissue was triturated gently in DMEM buffer using a series of fire-polished Pasteur pipettes (inner diameter 600, 300, and 150 μm) and the DMEM/neuron suspension was placed in a recording chamber on a fixed-stage fluorescence microscope (Zeiss Axio Examiner D1) for 10 min, followed by continuous superfusion at 1 mL/min in a standard artificial cerebrospinal solution (in mmol/L): 125 NaCl, 3 KCl, 1.2 KH_2_PO_4_, 1.2 MgSO_4_, 25 NaHCO_3_, 10 D-glucose, and 2 CaCl_2_, saturated with 95% O_2_–5% CO_2_, pH 7.4, 300 mOsm) at ~31°C. Individual EGFP fluorescent cells were aspirated into pipettes containing 10× Reverse Transcription buffer and RNaseOUT (Superscript III, Invitrogen), and expelled (~1 μL) into a sterile tube containing Deoxynucleotide Triphosphates, bovine serum albumin (BSA), RNaseOUT, MgCl_2_, oligo-dT, and random hexamers. The pre-RT mixture was incubated at 65°C, first-strand cDNA was synthesized with Superscript III reverse transcriptase, RNA was digested with RNase H, and cDNA was stored at ‒20°C. Two rounds of conventional PCR (kits purchased from Vazyme Biotech Co., Ltd, China) used pairs of gene-specific primer pairs. Primers were prepared for GAPDH (forward: GCAAATTCAACGGCACAGTCAAGG; reverse: TCTCGTGGTTCACACCC ATCACAA), PNMT (forward: CGGGCTTTGCATCACATCAC; reverse: GGACCTCGTAACCA CCAAGG), and TH (forward: GTCTCAGAGCAGGATACCAAGC; reverse: CTCTCCTCGAATACC ACAGCC). We included a no-template negative control (H_2_O) for each experiment; amplification of GAPDH mRNA served as a positive control.

### Immunohistochemical staining

The animals were deeply anesthetized with urethane (1.8 g/kg, i.p.) and transcardially perfused with chilled saline, followed by paraformaldehyde (PFA, 4% in PBS). After decapitation, the brainstem was post-fixed for 24 h in 4% PFA at 4°C and subsequently transferred to a 30% PBS-buffered sucrose solution until the it was saturated (24–36 h). Coronal sections were cut at 25 μm on a freezing microtome (CM1950; Leica Microsystems, Germany). The sections were blocked in 5% bovine serum albumin (BSA) in 0.25% Triton X-100 in PBS) for 30 min at room temperature (23–24°C), followed by incubation with primary antibodies in 2% BSA–PBS overnight at 4°C. Then the sections were washed with PBS (3 × 5 min) and incubated with fluorescent secondary antibodies at room temperature for 1 h. All rinses and incubations were done over a shaker at low speed. After rinsing with PBS (3 × 5 min), the sections were mounted on slides with Vectashield Antifade Mounting Medium (Vector Laboratories, Burlingame, CA, USA) for visualization. Images were captured using a laser-scanning confocal microscope (LSM 800, Carl Zeiss, Germany) and processed with ZEN software (Zeiss, Germany). Cells were manually counted in confocal images.

The primary antibodies used were as follows: chicken anti-GFP (dilution 1:2000, catalog# ab13970, RRID: AB_300798, Abcam), rabbit anti-TH (1:1000, EMD Millipore, catalog# AB152), rabbit anti-neuronal nitric oxide synthase (nNOS, 1:500, Cell Signaling Technology, catalog# PA1-033, RRID: AB_325021), rabbit anti-FOXP2 (1:1000, Abcam, catalog# ab16046, RRID: AB_2813765). The fluorophore-conjugated secondary antibodies used were: Cy™3 AffiniPure goat anti-rabbit IgG (H+L) (1:500, Jackson ImmunoResearch Laboratories, catalog# 111-165-003, RRID: AB_2338000), goat polyclonal secondary antibody to chicken IgY-H&L (Alexa Fluor® 488) (1:1000, Abcam, catalog# ab150169, RRID: AB_2636803).

To examine the distribution pattern of NTS^PNMT^ neurons, the immunohistochemical expression of EGFP was assessed 4 weeks after bilateral injections of AAV-hSyn-DIO-EGFP into the NTS of PNMT-Cre mice. EGFP-expressing NTS neurons were counted in 10 coronal sections from each mouse as described above. TH immunoreactivity in NTS neurons expressing EGFP was also checked.

### Statistics

All data were imported into Prism 9 (GraphPad, RRID: SCR_002798) for statistical analyses. All data are presented as the mean ± SEM. Differences between two groups were assessed using a two-sided Student’s *t*-test, where *P* <0.05 was considered significant and *P* >0.05 was considered non-significant. Multiple groups were compared using analysis of variance (ANOVA, one or two-way), followed by appropriate *post hoc* tests as indicated in the text.

## Results

### Histomolecular Validation of PNMT Expression in the NTS

**Fig. 1:**
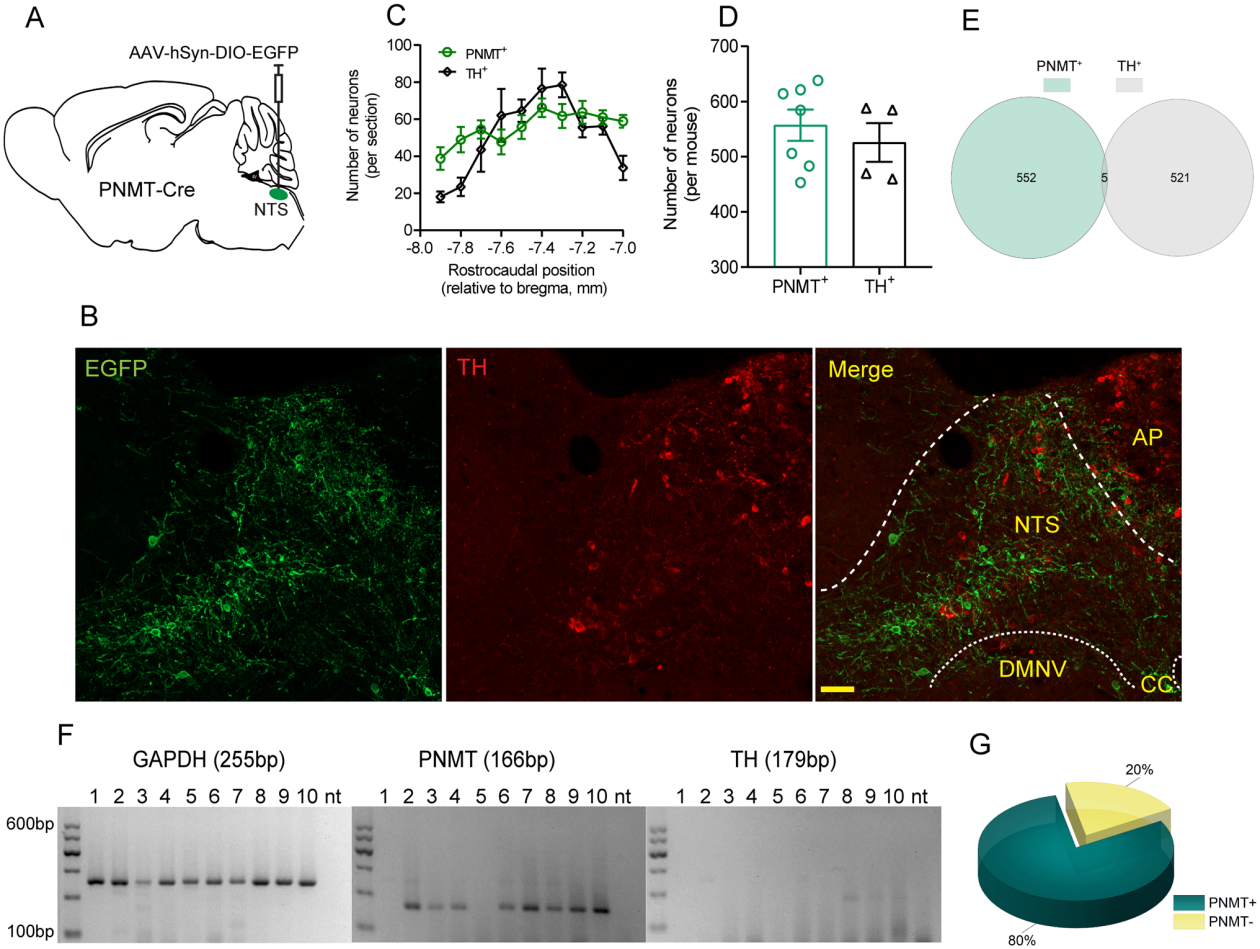
Validation of PNMT expression in NTS neurons. **A** Schematic of delivery of a Cre-inducible virus into the NTS of PNMT-Cre mice. **B** Confocal images showing TH immunoreactivity of EGFP-expressing neurons. Scale bar, 50 μm. **C** Rostrocaudal distribution of PNMT- and TH-immunoreactive neurons. **D** The total number of PNMT^+^ and TH^+^ neurons was calculated based on panel **C**. **E** A small number of neurons is immunoreactive to both PNMT and TH. **F** Single-cell RT-PCR from a representative sample of EGFP-expressing dissociated NTS neurons indicates the expression of PNMT in 8 of 10 cells tested (lanes 2–4 and 6–10), with no evidence for TH. The positive control GAPDH transcript was universally detectable, and no template negative controls were included (nt lane). **G** Quantification of single-cell RT-PCR data reveals PNMT expression in 80% of EGFP-expressing NTS neurons (*n =* 20). TH, tyrosine hydroxylase; DMNV, dorsal motor nucleus of the vagus; AP, area postrema; CC, central channel.

Early studies have provided morphological evidence of PNMT immunoreactivity in the NTS [23, 24]. Here, we further characterized the PNMT distribution pattern in the NTS in a PNMT-Cre mouse line. To that end, a Cre-inducible virus was injected into the NTS (Fig. 1A), followed by immunohistochemical assessment of EGFP expression. As shown in Fig. 1, EGFP-labeled neurons spanned the caudal, intermediate, and rostral parts of the NTS, with a greater number of neurons in the intermediate and rostral regions (Fig. 1B, C). To further reveal the catecholaminergic identity, we determined whether EGFP-labeled neurons were immunoreactive to TH, a general marker of noradrenergic neurons. Interestingly, very few EGFP-expressing neurons were immunoreactive to TH (Fig. 1B, E). Based on bilateral cell counts (Fig. 1C), the total number of EGFP-immunoreactive neurons per mouse was 557 ± 28 (*n =* 7 mice) and that of TH-immunoreactive neurons was 526 ± 35 (*n =* 4 mice) (Fig. 1D). Furthermore, we used an scPCR reaction to validate the presence of PNMT and TH in acutely dissociated fluorescent NTS neurons. The gel images shown in Fig. 1F revealed PNMT amplicons in 8 of 10 individual neurons expressing EGFP (lanes 2–4 and 6–10), without detection of the TH amplicon. Overall, we found PNMT expression in 80% of the EGFP-labeled NTS neurons tested by scPCR (*n =* 16 of 20; Fig. 1G). Almost no TH expression was detected in both scPCR and immunohistochemical data. Together, these data suggest that PNMT expression is detectable in the majority of EGFP-labeled NTS neurons and this population exhibits almost no TH immunoreactivity.

### Photostimulation of NTS^PNMT^ Neurons has Variable Effects on BP

**Fig. 2:**
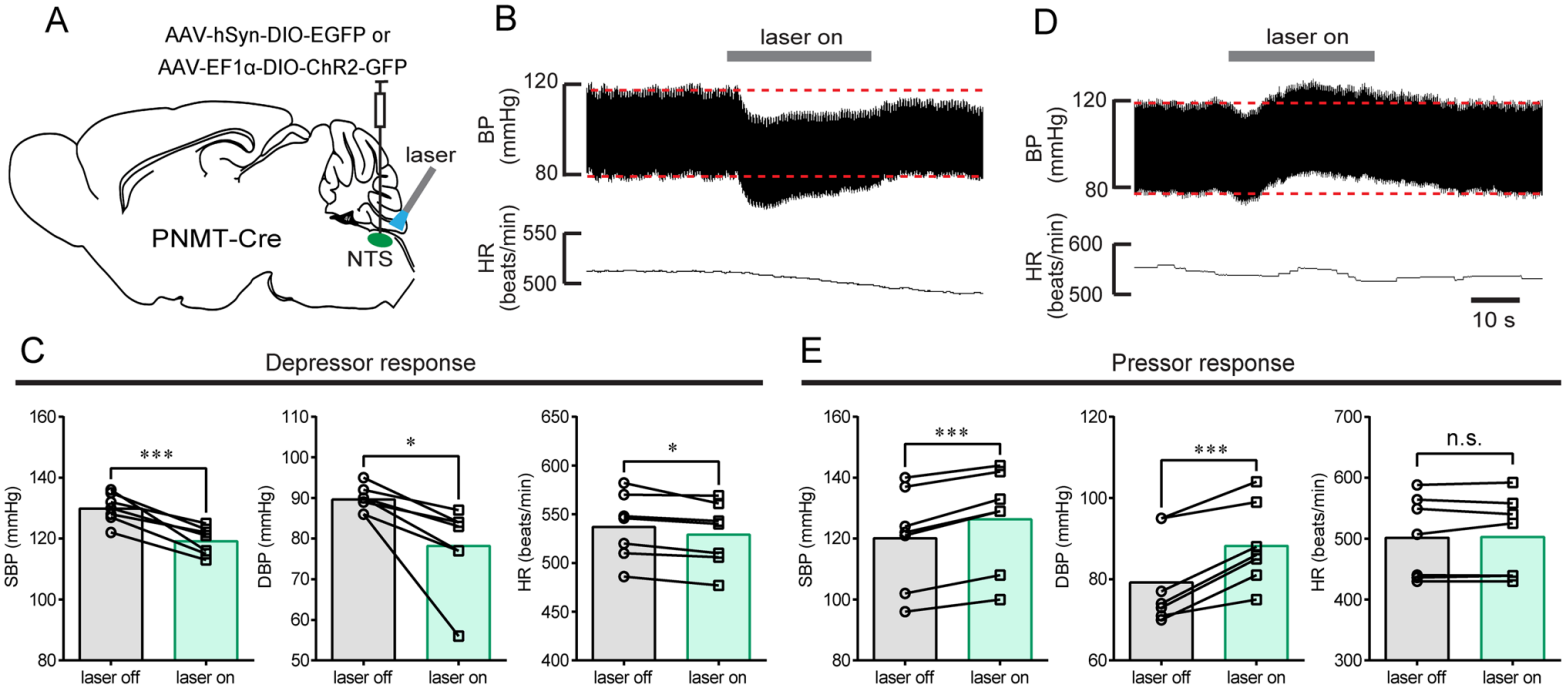
Optogenetic stimulation of NTS^PNMT^ neurons has variable effects on BP and HR. **A** Schematic of the optogenetics strategy by injections of a virus encoding ChR2 into the NTS. **B** Typical traces of a depressor response caused by photostimulation. **C** Group data (*n =* 7) indicates that NTS^PNMT^ neuron stimulation increases SBP and DBP but decreases HR. **D** Typical traces of a pressor response evoked by photostimulation. **E** Group data (*n =* 7) indicating photostimulation-induced increases in SBP and DBP, with unchanged HR. ^*^P <0.05, ^***^P <0.001, two-tailed paired *t*-test. ns, no significant difference.

Although catecholaminergic NTS neurons have been implicated in the control of BP, the neural circuits and subset of these neurons remain to be comprehensively elucidated. To examine the contribution of NTS^PNMT^ neurons to cardiovascular activity, we used an optogenetic approach to selectively stimulate these neurons in anesthetized PNMT-Cre mice. Four weeks after injections of AAV9-EF1α-DIO-ChR2-GFP into the NTS (Fig. 2A), immunohistochemical data revealed ChR2 expression in a similar manner (Fig. 1B). Optogenetic stimulation of NTS^PNMT^ neurons had variable effects on BP, with a depressor response in 7 anesthetized mice (SBP: 130 ± 2 *vs* 119 ± 2 mmHg, laser OFF *vs* laser ON, *P* <0.001; DBP: 90 ± 1 *vs* 78 ± 4 mmHg, laser OFF *vs* laser ON, *P* <0.05; Fig. 2B, C) and a pressor response in the other 7 anesthetized mice (SBP: 120 ± 6 *vs* 126 ± 6 mmHg, laser OFF *vs* laser ON; DBP: 79 ± 4 *vs* 88 ± 4 mmHg, laser OFF *vs* laser ON; *P* < 0.001 for both SBP and DBP; Fig. 2D, E). There was a moderate decrease in HR in mice with the depressor response (537 ± 13 *vs* 529 ± 12 beats/min, laser OFF *vs* laser ON, *P* <0.05, Fig. 2C), whereas no significant change in HR was observed in those with the pressor response (502 ± 25 *vs* 503 ± 25 beat/min, laser OFF *vs* laser ON, *P* >0.05, Fig. 2E).

In control experiments using PNMT-Cre mice (*n =* 10) subjected to injections of a virus devoid of ChR2 in the NTS; illumination of the NTS led to no significant change in both BP (SBP: 119 ± 1 *vs* 120 ± 1 mmHg, laser OFF *vs* laser ON; DBP: 89 ± 1 *vs* 89 ± 1 mmHg, laser OFF *vs* laser ON; *P* >0.05 for both SBP and DBP, paired *t*-test) and HR (524 ± 11 *vs* 525 ± 10 beats/min, laser OFF *vs* laser ON, *P* >0.05 by paired *t*-test). Therefore, we ascribed the above cardiovascular effects to NTS^PNMT^ neuron stimulation rather than an optogenetic side-effect.

### NTS^PNMT^ Neurons Projecting to the PVN and LPBN are Responsible for the Depressor Response

**Fig. 3:**
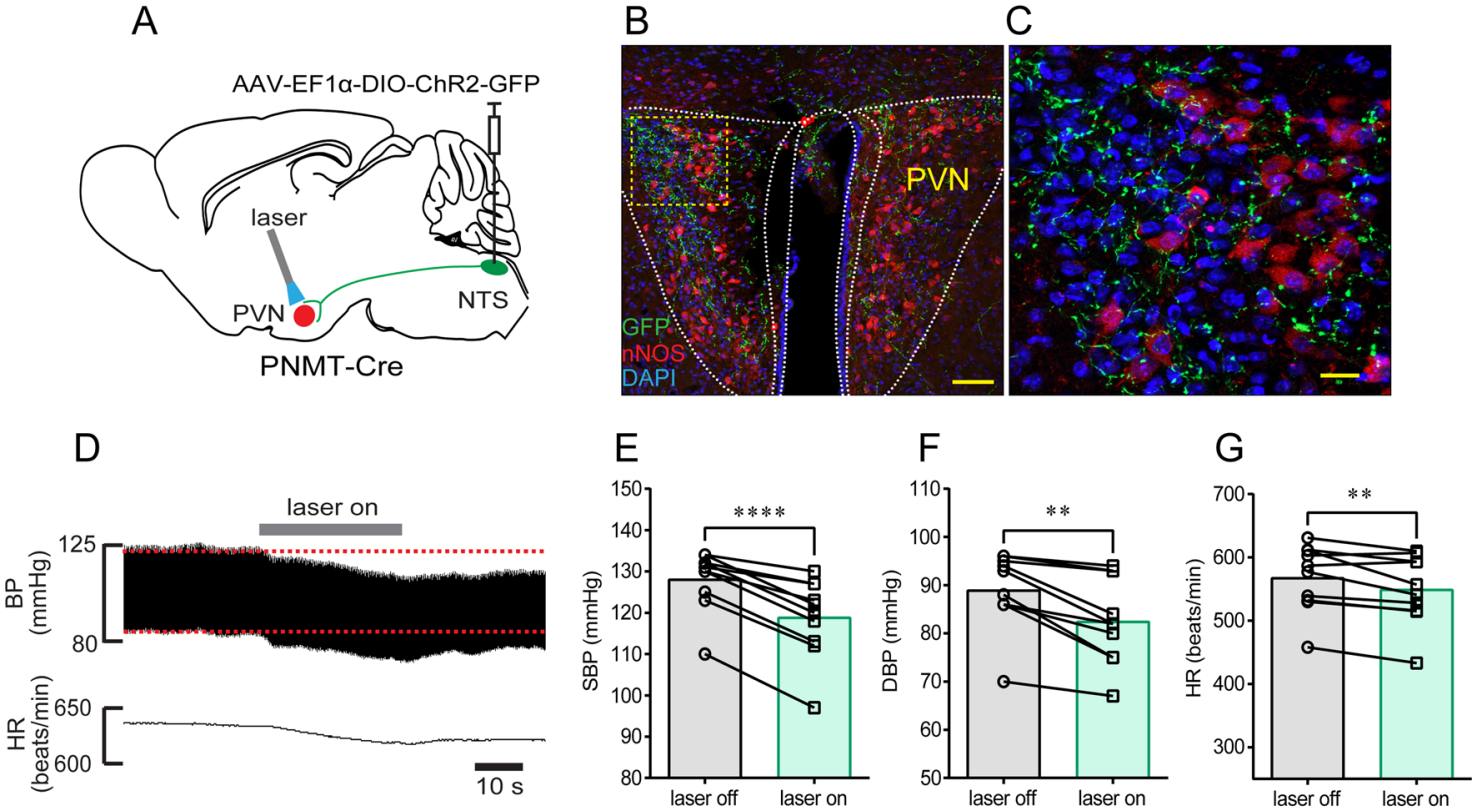
Photostimulation of NTS^PNMT^ neurons projecting to the PVN induces a decrease in BP. **A** Schematic of an optogenetic strategy for PVN-projecting NTS^PNMT^ neuron stimulation. **B** Confocal image showing the distribution of the axons (green) of NTS^PNMT^ neurons projecting to the PVN. Some PVN neurons are immunoreactive to nNOS (red). Scale bar, 50 μm. **C** Confocal image enlarged from the region denoted by the square in B. Scale bar, 20 μm. **D** Original traces demonstrating that illumination of the axons of NTS^PNMT^ neurons projecting to the PVN generate a decrease in both BP and HR. **E–G** Statistical analyses of group data. *n =* 10, ^**^P <0.01, ^****^P <0.0001, paired *t*-test. PVN, paraventricular nucleus of the hypothalamus; nNOS, neuronal nitric oxide synthases.

Next, we sought to determine the circuit mechanism underlying the depressor responses described above. The PVN is an important target for the central control of BP and consists of pre-sympathetic neurons [3, 25]. Based on this fact, we asked if activation of NTS^PNMT^ neurons projecting to the PVN elicited the depressor response. Following injections of the virus encoding ChR2 into the NTS (Fig. 3A), ChR2-GFP was assessed using immunohistochemical assays. Because PVN neurons expressing nNOS contribute to the regulation of BP [26], the corresponding antibody was used to label this type of PVN neuron. As shown in Fig. 3B, C, the axons of NTS^PNMT^ neurons were highly distributed around the PVN neurons including nNOS-expressing neurons. Illumination of the axons of NTS^PNMT^ neurons in the PVN of anesthetized mice (*n =* 10) generated a remarkable decrease in both BP (SBP:128 ± 2 *vs* 119 ± 3 mmHg, laser OFF *vs* laser ON, *P* <0.0001; DBP: 89 ± 3 *vs* 83 ± 3 mmHg, laser OFF *vs* laser ON; *P* <0.01 for both SBP and DBP; Fig. 3E, F) and HR (568 ± 17 *vs* 549 ± 17 beats/min, laser OFF *vs* laser ON, *P* <0.01, Fig. 3G).

**Fig. 4:**
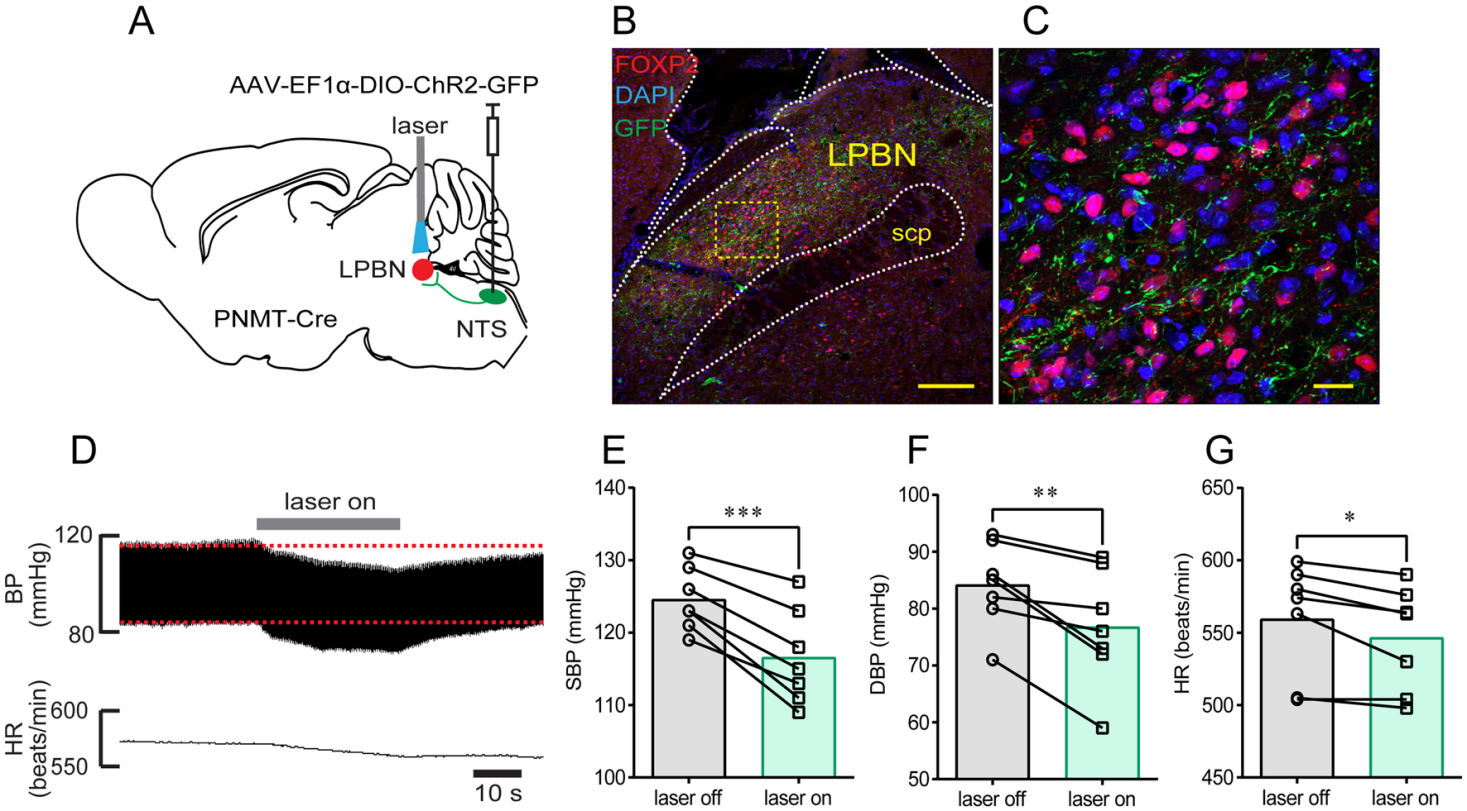
Photostimulation of NTS^PNMT^ neurons projecting to the LPBN reduces both BP and HR. **A** Schematic of an optogenetic strategy. **B** Confocal image showing the distribution of axons (green) of NTS^PNMT^ neurons projecting to the LPBN. FOXP2 (red) is a relatively specific marker to label LPBN neurons. Scale bar, 50 μm. **C** Confocal image enlarged from the region denoted by the square in B. Scale bar, 20 μm. **D** Representative traces showing that photostimulation of the axons of NTS^PNMT^ neurons projecting to the LPBN cause a decrease in both BP and HR. **E–G** Statistical analyses of group data. *n =* 7, ^*^P <0.05, ^**^P <0.01, ^***^P <0.001, paired *t*-test. LPBN, lateral parabrachial nucleus; FOXP2, Forkhead box protein P2; scp, superior cerebellar peduncle.

Prior studies demonstrated a major role of the LPBN in regulating cardiovascular function [27], as well as anatomical evidence showing connections between the LPBN and the NTS [28]. In the present study, we checked the projections from the NTS^PNMT^ neurons to the LPBN by injections of an anterograde tracing virus into the NTS (Fig. 4A). According to a prior report showing the contribution of LPBN neurons expressing FOXP2 to the regulation of BP [29], we used FOXP2 to label putative neurons associated with controlling cardiovascular activity. Confocal images revealed the presence of numerous axons of NTS^PNMT^ neurons in the LPBN and quite a few FOXP2-expressing neurons were wrapped by axons (Fig. 4B, C). Illumination of the LPBN in anesthetized PNMT-Cre mice (*n =* 7) markedly decreased SBP (125 ± 2 *vs* 117 ± 2 mmHg, laser OFF *vs* laser ON, *P* <0.001, Fig. 4E), DBP (84 ± 3 *vs* 77 ± 4 mmHg, laser OFF *vs* laser ON, *P* <0.01, Fig. 4F) and HR (559 ± 14 *vs* 546 ± 14 beats/min, laser OFF *vs* laser ON, *P* <0.05, Fig. 4G). Collectively, activation of NTS^PNMT^ neurons projecting to the LPBN produced a depressor response similar to the above effect found in the PVN.

### Stimulation of NTS^PNMT^ Neurons Projecting to the Ventrolateral Medulla Generates Opposing Cardiovascular Effects

**Fig. 5:**
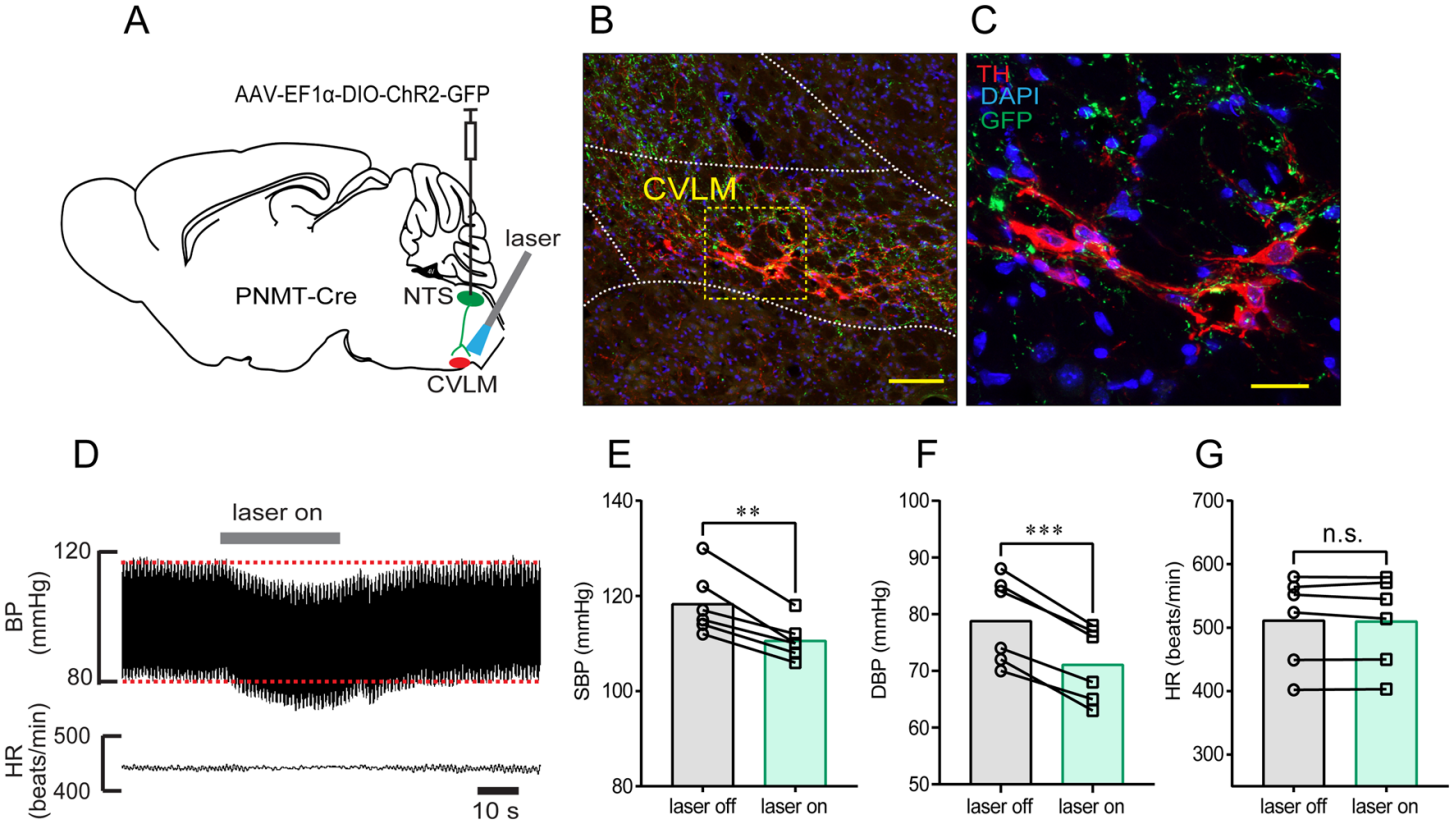
Optogenetic stimulation of NTS^PNMT^ neurons projecting to the CVLM lowers BP. **A** Schematic of a strategy of optogenetic stimulation. **B** Confocal image showing the distribution of axons (green) of NTS^PNMT^ neurons projecting to the CVLM. The hallmark of CVLM is indicated by staining with TH (red). Scale bar, 50 μm. **C** Confocal image enlarged from the region denoted by the square in B. Scale bar, 20 μm. **D** Typical traces showing a decrease in BP but not HR when illuminating the CVLM. **E–G** Statistical analyses of group data. *n =* 6, ^**^P <0.01, ^***^P <0.001, paired *t*-test.

Ventrolateral medulla neurons play a crucial role in the autonomic regulation of cardiovascular activity. CVLM neurons are the essential component of the baroreflex control of BP. Our anatomical evidence also supported the connection between the NTS and CVLM regions [20]. Here, we sought to dissect the anatomical and functional circuits of NTS^PNMT^ neurons projecting to the CVLM. First, we injected the virus encoding ChR2 into the NTS (Fig. 5A), and this was followed by immunohistochemical detection of axons of NTS^PNMT^ neurons in the CVLM that was generally identified by TH immunostaining. As shown in Fig. 5, both axon terminals and TH-immunoreactive neurons were observed in the CVLM (Fig. 5B, C). Illumination of the CVLM of anesthetized mice (*n =* 6) produced a moderate decrease in SBP (118 ± 3 *vs* 111 ± 2 mmHg, laser OFF *vs* laser ON, *P* <0.01, Fig. 5D, E) and DBP (79 ± 3 *vs* 71 ± 3 mmHg, laser OFF *vs* laser ON, *P* <0.001, Fig. 5D, F). However, HR was unchanged before and after photostimulation (512 ± 29 *vs* 510 ± 29 beats/min, laser OFF *vs* laser ON, *P* >0.05, Fig. 5G). Hence, activation of NTS^PNMT^ neurons projecting to the CVLM is responsible for the depressor response.

**Fig. 6:**
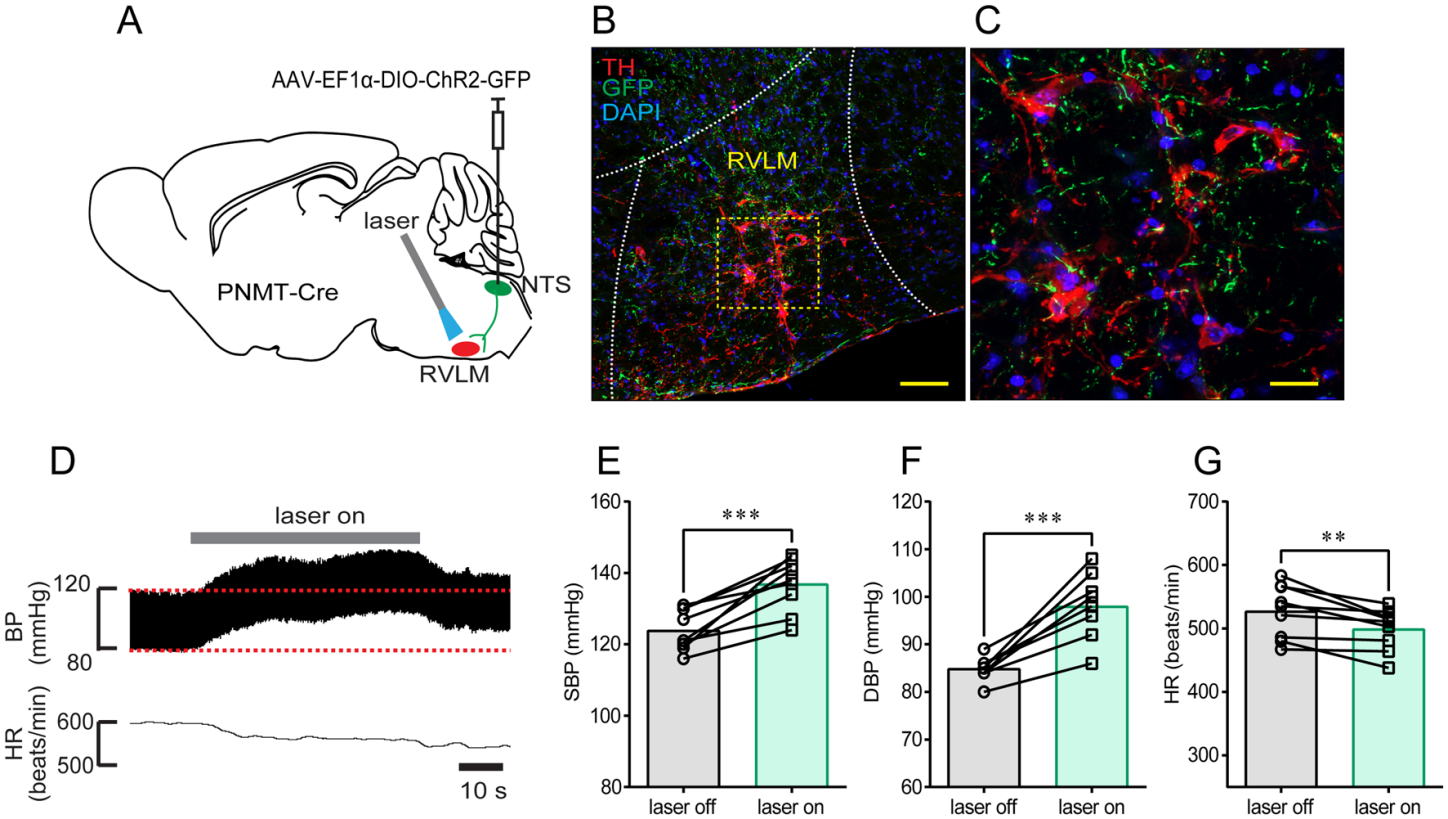
Illumination of axon terminals of NTS^PNMT^ neurons projecting to the RVLM causes a pressor response. **A** Schematic of an optogenetics strategy. **B** Confocal image showing the distribution of the axons (green) of RVLM-projecting NTS^PNMT^ neurons. TH (red) labels C1 neurons in the RVLM. Scale bar, 50 μm. **C** Confocal image enlarged from the region denoted by the square in B. Scale bar, 20 μm. **D** Illumination of the RVLM induces a robust increase in BP, with a decrease in HR. **E–G** Statistical analyses of group data. *n =* 9, ^**^P <0.01, ^***^P <0.001, paired *t*-test.

RVLM neurons participate in the sympathetic control of BP. To test whether NTS^PNMT^ neurons project to the RVLM and whether these neurons contributed to the modulation of BP, a similar optogenetics protocol was applied as described above (Fig. 6A). Immunohistochemical detection revealed that axons of NTS^PNMT^ neurons were densely distributed in the RVLM region; The contour of the RVLM was marked by TH immunoreactivity (Fig. 6B, C). Photostimulation of axons in the RVLM of anesthetized PNMT-Cre mice (*n =* 9) elicited a rapid increase in SBP (124 ± 2 *vs* 137 ± 2 mmHg, laser OFF *vs* laser ON, *P* <0.001, Fig. 6E) and DBP (85 ± 1 *vs* 98 ± 2 mmHg, laser OFF *vs* laser ON, *P* <0.001, Fig. 6F), but a decrease in HR (527 ± 14 *vs* 499 ± 11 beats/min, laser OFF *vs* laser ON, *P* <0.01, Fig. 6G).

**Fig. 7:**
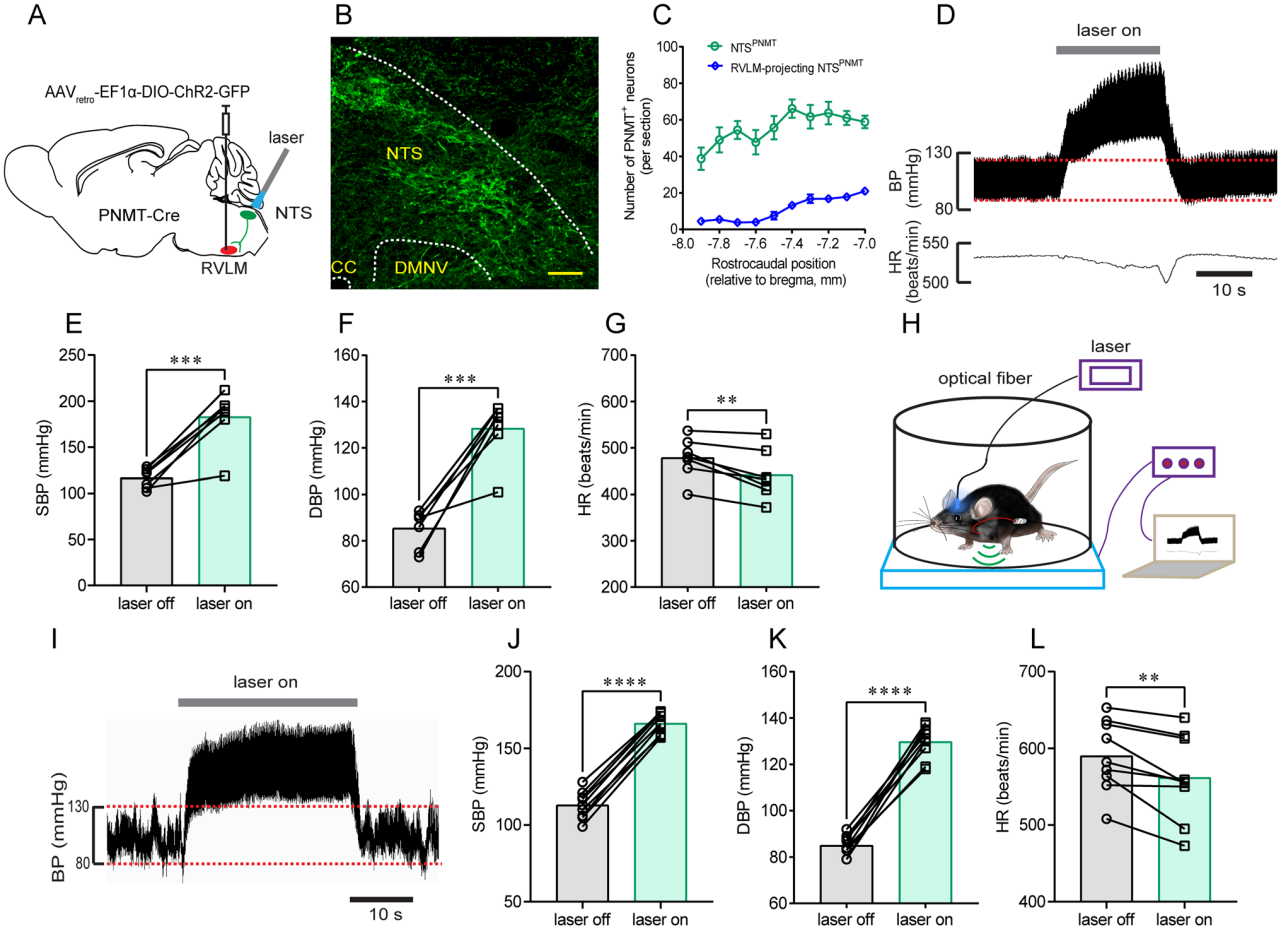
Illumination of somata of NTS^PNMT^ neurons retrogradely labeled from the RVLM markedly elevates BP. **A** Schematic of an optogenetics strategy *via* delivery of a retrograde virus into the RVLM. **B** Identification of retrogradely-labeled neurons in the NTS. Scale bar, 50 μm. **C** Rostrocaudal distribution of NTS^PNMT^ neurons projecting to the RVLM (blue line). For comparison, all the NTS^PNMT^ neurons are present as shown in Fig. 1C. **D** Representative traces of a pressor response evoked by photostimulation. **E–G** Statistical analyses of group data (*n =* 7). **H** Schematic of the combined application of radio telemetry recording of BP and optogenetic stimulation. **I** Typical traces of a pressor response elicited by illumination of the NTS in an awake mouse. **J–L** Statistical analyses of pooled data (*n =* 9). ^**^P <0.01, ^***^P <0.001, ^****^P <0.0001, paired *t*-test.

To further verify the circuits of this pressor response, we injected a retrograde tracing virus into the RVLM (Fig. 7A). Immunohistochemical validation located GFP-expressing NTS neurons (Fig. 7B), and these neurons were then bilaterally counted in coronal sections based on rostrocaudal position relative to bregma (Fig. 7C). Illumination of the NTS in anesthetized PNMT-Cre mice (*n =* 7) produced a robust increase in SBP (117 ± 4 *vs* 183 ± 11 mmHg, laser OFF *vs* laser ON, *P* <0.001, Fig. 7E) and DBP (85 ± 3 *vs* 128 ± 5 mmHg, laser OFF *vs* laser ON, *P* <0.001, Fig. 7F), and a decrease in HR (479 ± 16 *vs* 442 ± 20 beats/min, laser OFF *vs* laser ON, *P* <0.01, Fig. 7G). Moreover, the cardiovascular effects of NTS^PNMT^ neurons projecting to the RVLM were also assessed using a radio telemetry system in awake PNMT-Cre mice (*n =* 9, Fig. 7H). Similarly, illumination of the NTS resulted in a vigorous increase in BP (SBP: 113 ± 3 *vs* 166 ± 2 mmHg, laser OFF *vs* laser ON, *P* <0.0001; DBP: 85 ± 1 *vs* 130 ± 2 mmHg, laser OFF *vs* laser ON, *P* <0.0001; Fig. 7I–K) and a decrease in HR (590 ± 16 *vs* 562 ± 18 beats/min, laser OFF *vs* laser ON, *P* <0.01, Fig. 7L), suggesting that NTS^PNMT^ neurons projecting to the RVLM are responsible for the pressor response. Together, activation of NTS^PNMT^ neurons projecting to the CVLM and the RVLM had opposing effects on BP.

### Ablation of NTS^PNMT^ Neurons Impairs the Arterial Baroreflex

**Fig. 8:**
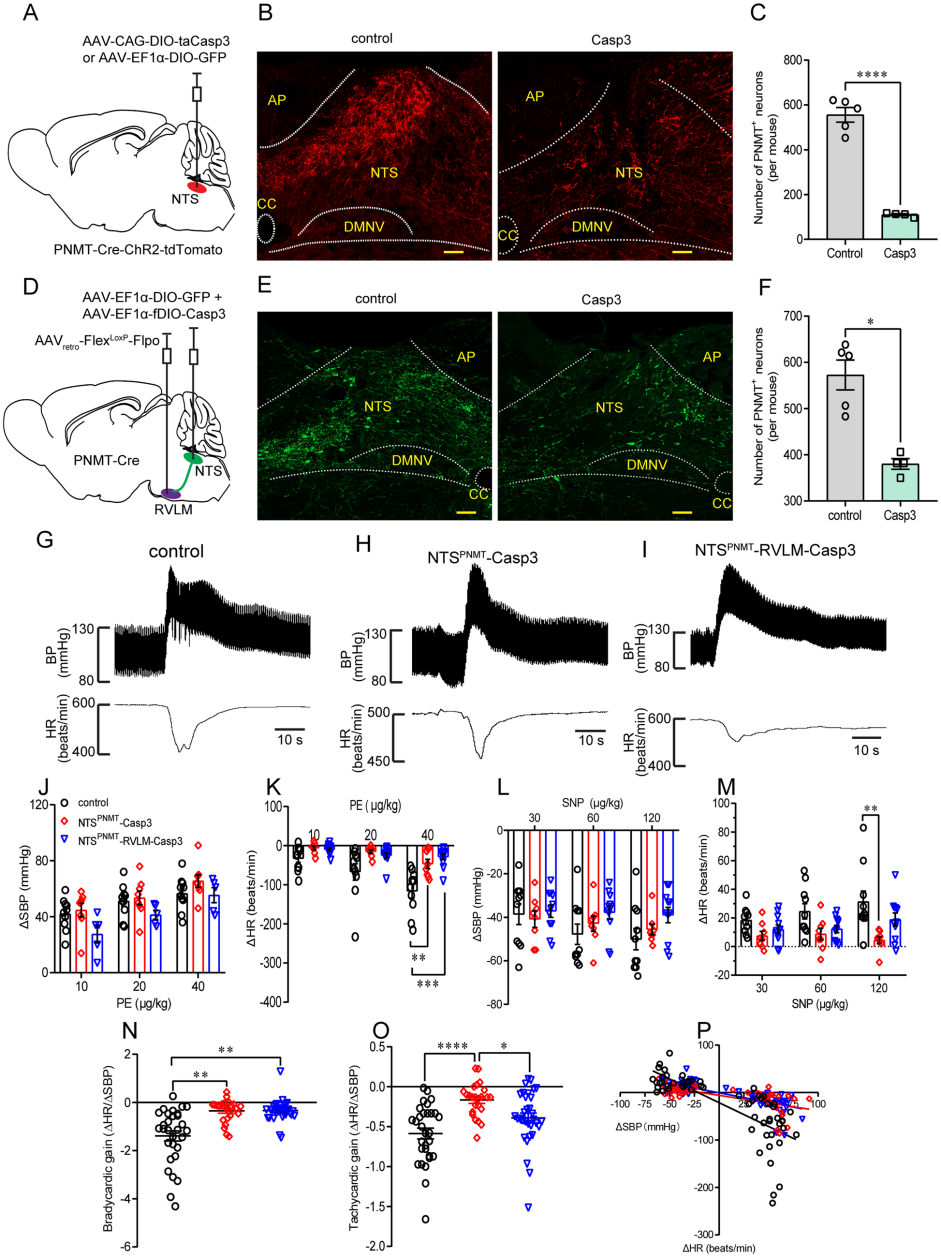
Ablation of NTS^PNMT^ neurons impairs the baroreflex. **A** Schematic of a genetic ablation strategy *via* injections of a virus encoding the Casp3 gene into the NTS in PNMT-Cre-ChR2-tdTomato mice. Control mice were injected with a virus devoid of the Casp3 gene. **B** Confocal images showing the number of NTS^PNMT^ neurons is lower in Casp3-injected mice than in the control. Scale bar, 50 μm. **C** Quantitative analysis of neuronal ablation (*n =* 5 for control, *n =* 4 for Casp3; unpaired *t*-test). **D** Schematic of a strategy for the selective ablation of NTS^PNMT^ neurons projecting to the RVLM in PNMT-Cre mice. **E** Confocal images showing GFP-labelled neurons are fewer in the Casp3-injected mouse than in the control. Scale bar, 50 μm. **F** Quantitative analysis of neuronal ablation (*n =* 5 for control, *n =* 4 for Casp3; Mann-Whitney test). **G**–**I** Typical traces showing the responses of BP and HR to intravenous injection of PE (40 µg/kg, i.v.) in the three phenotypes. **J, K** The mean changes in ∆SBP and ∆HR in responses to intravenous injections of different doses of PE (10, 20, and 40 µg/kg; two-way ANOVA with Tukey’s multiple comparisons test). **L, M** The mean change in ∆SBP and ∆HR in responses to intravenous injection of SNP (30, 60, and 120 µg/kg; two-way ANOVA with Tukey’s multiple comparisons test). **N, O** Bradycardia and tachycardiac gains of NTS^PNMT^-Casp3 mice are significantly lower than in the control group. Bradycardia rather than tachycardiac gain is significantly decreased in mice with ablation of NTS^PNMT^ neurons (Kruskal-Wallis test followed by Dunn's multiple comparisons test). **P** The baroreflex curve showing the individual data points for the change in ∆HR in response to induced changes in ∆SBP. Linear regression analysis shows a significant difference between groups. *n =* 10 mice/30 responses for control mice without loss of NTS^PNMT^ neurons, *n =* 8 mice/24 responses for mice subjected to ablation of NTS^PNMT^ neurons, *n =* 11 mice/33 responses for mice subjected to ablation of RVLM-projecting NTS^PNMT^ neurons. ^*^P <0.05, ^**^P <0.01, ^***^P <0.001, ^****^P <0.0001.

To determine whether NTS^PNMT^ neurons contribute to regulation of the baroreflex, loss-of-function experiments were carried out *via* ablation of these neurons. To that end, a genetic method was used to eliminate NTS^PNMT^ neurons by injections of a Cre-inducible virus encoding Casp3 into the NTS of PNMT-Cre-tdTomato mice (Fig. 8A) as reported previously [17, 20], followed by immunohistochemical validation of the ablation efficiency. The number of tdTomato-labelled neurons was remarkably reduced in Casp3-injected mice relative to the control mice (556 ± 33 *vs* 110 ± 4, control *vs* Casp3; *n =* 5 for control, *n =* 4 for Casp3, *P* <0.0001; Fig. 8B, C). Thus, ~80% of NTS^PNMT^ neurons were ablated. To selectively ablate NTS^PNMT^ neurons projecting to the RVLM, the Cre/LoxP and Flpo/FRT double system-based viruses were each injected into the NTS and the RVLM in PNMT-Cre mice (Fig. 8D). According to the cell counts, the number of GFP-labelled neurons was significantly reduced in Casp3-injected mice relative to the control mice (572 ± 32 *vs* 380 ± 11, control *vs* Casp3; *n =* 5 for control, *n =* 4 for Casp3, *P* <0.05, Fig. 8E, F). Hence, ~34% of NTS^PNMT^ neurons were destroyed.

Following the ablation of NTS^PNMT^ neurons, we assessed the arterial baroreflex in three groups of mice: those (*n =* 10) without loss of NTS^PNMT^ neurons, those (*n =* 8) subjected to ablation of NTS^PNMT^ neurons, and those (*n* = 11) subjected to ablation of NTS^PNMT^ neurons projecting to the RVLM. Different doses of PE (10, 20, or 40 μg/kg) and SNP (30, 60, or 120 μg/kg) were intravenously injected to evoke the pressor and depressor responses in anesthetized mice. Fig. 8G–I shows representative traces of the PE (40 μg/kg)-evoked pressor effect and bradycardic response in the three groups of mice. Group data analysis showed that injections of different doses of PE induced similar rises in BP in all three groups (Fig. 8J). However, the bradycardia evoked by a high dose of PE (40 μg/kg) was remarkably depressed in the two ablation groups relative to the control (ΔHR: −116 ± 18 *vs* −47 ± 12 beats/min, control *vs* NTS^PNMT^-Casp3, *P* <0.01; −116 ± 56 *vs* −29 ± 25 beats/min, control *vs* NTS^PNMT^- RVLM-Casp3, *P* <0.001; Fig. 8K). On the other hand, no significant difference in the SNP-induced BP decrease was found among the three groups (Fig. 8L). SNP-evoked tachycardia was also similar among the three groups, except that a high dose of SNP (120 μg/kg) induced greater tachycardic depression in mice subjected to ablation of NTS^PNMT^ neurons relative to the control (ΔHR: 31 ± 7 *vs* 5 ± 3 beats/min, control *vs* NTS^PNMT^-Casp3, *P* <0.01; Fig. 8M). Therefore, ablation of both NTS^PNMT^ neurons and those projecting to the RVLM significantly reduced the bradycardic gain (−1.39 ± 0.21 *vs* −0.35 ± 0.09, control *vs* NTS^PNMT^-Casp3, *P* <0.0001; −1.39 ± 0.21 *vs* −0.34 ± 0.08, control *vs* RVLM-NTS^PNMT^-Casp3, *P* <0.0001; Fig. 8N, P). However, the tachycardic gain was considerably impaired in mice subjected to ablation of NTS^PNMT^ neurons rather than those projecting to the RVLM (−0.59 ± 0.07 *vs* −0.17 ± 0.04, control *vs* NTS^PNMT^-Casp3, *P* <0.0001, Fig. 8O, P). Together, ablation of both NTS^PNMT^ neurons and those projecting to the RVLM impaired the baroreflex.

## Discussion

In the present study, we demonstrated that optogenetic stimulation of NTS^PNMT^ neurons had variable effects on BP and HR in anesthetized PNMT-Cre mice. Stimulation of NTS^PNMT^ neurons projecting to the PVN, LPBN, and CVLM significantly reduced BP. However, stimulation of those projecting to the RVLM in both anesthetized and awake mice induced a remarkable pressor response and bradycardia. Ablation of NTS^PNMT^ neurons significantly impaired both bradycardic and tachycardic gains, whereas ablation of those solely projecting to the RVLM diminished bradycardic but not tachycardic gain, suggesting that NTS^PNMT^ neurons contribute to maintaining the baroreflex.

### Molecular Phenotypes of Catecholaminergic NTS Neurons

The central adrenergic neurons were mainly located in the RVLM (C1 neurons), the NTS (C2 neurons), and the rostral part of the dorsomedial medulla (C3 neurons). Most of the C1 neurons were shown to be double- or triple-labeled with TH, DβH, and PNMT, whereas some cells lacked DβH immunoreactivity; a small number of cells was immunoreactive for only one of the three enzymes [30]. This differential expression pattern suggests that a proportion of C1 neurons may not express all the enzymes responsible for adrenaline synthesis. More recently, catecholaminergic NTS neurons have been implicated in many physiological processes, including the regulation of hypoglycemic hunger in TH-Cre mice [31], blood glucose levels [13], and anorexia [12] in DβH-Cre mice. These studies focused on the functional phenotypes of catecholaminergic NTS neurons but lacked evidence that they released the catecholamine, and if so, which (dopamine, noradrenaline, adrenaline, or all) and where (varicosities or axon terminals).

Previous studies have shown the presence of PNMT-immunoreactive neurons in the NTS [32, 33], while the co-expression pattern of TH, PNMT, and DβH remains poorly understood. Here, a PNMT-Cre mouse line was for the first time used to address these questions; the scPCR analysis revealed that PNMT was present in ~80% of fluorescently-labeled NTS neurons, confirming the reliability of this PNMT-Cre mouse line in the selective targeting adrenergic neurons. Hence, we assumed that fluorescently-labeled NTS^PNMT^ neurons from the PNMT-Cre mouse line belonged to the adrenergic C2 group. Both the scPCR analysis and immunohistochemical detection revealed that the majority of fluorescently-labeled neurons exhibited no TH immunoreactivity, inconsistent with the high co-expression pattern of TH and PNMT in C1 neurons. Since TH, DβH, and PNMT are key enzymes during the cascade of synthesis of dopamine, noradrenaline, and adrenaline, TH is presumably contained but actually absent in NTS^PNMT^ neurons based on the present study. This separate expression profile of TH and PNMT in the NTS has also been reported in the rat retina [34]. It could be that there is a non-classical pathway of catecholamine biosynthesis in the NTS. The mechanism responsible for the loss of TH immunoreactivity in NTS^PNMT^ neurons has not yet been investigated but needs to be unveiled in the future.

### Circuits Controlling the Depressor Response by NTS^PNMT^ Neurons

The application of state-of-the-art techniques (e.g., optogenetics) has advanced our understanding of specific neural circuits underlying certain behaviors and established novel therapies for various disorders based on spatiotemporally manipulated behavior-specific circuits. The NTS not only receives extensive afferent information ranging from visceral organs to higher brain areas, but also sends extensive efferents to control behavior and metabolism. Here, selective stimulation of NTS^PNMT^ neurons had variable effects on BP. Such changes in BP were derived by subtraction of the amplitude of pressor and depressor responses. We consider such differential responses are attributable to the following factors, including but not limited to the number of ChR2-transduced neurons, illumination location and area, and the number and neurochemical phenotypes of activated downstream neurons. Most importantly, such differential effects could highly rely on the recruitment of different neural circuits from the NTS to other brain regions.

It should be noted here that illumination invariably heats cells to probably produce changes in neural activity and behavior; a higher-power laser may cause severe damage to neurons. In the present study, to maximally reduce optogenetic deficiency, laser parameters were set based on similar experiments [17, 35]; control experiments were conducted in mice with injections of viruses devoid of ChR2 to assess the laser-induced heating effect on BP and HR; stimulation of NTS^PNMT^ neurons without ChR2 expression produced no significant cardiovascular effects.

Mutual projections between the NTS and the PVN have been demonstrated [36, 37]. King *et al*. found that TH-expressing NTS neurons projecting to the PVN are critical for hypoxic activation of the chemoreflex [38]; this TH-expressing subset is unlikely to be PNMT-positive based on the present findings. In addition, β1-adrenergic receptor signaling contributes to brain-derived neurotrophic factor-mediated regulation of BP by NTS catecholaminergic neurons projecting to the PVN. Here, we demonstrated that photostimulation of NTS^PNMT^ neurons projecting to the PVN caused a depressor response, revealing a neuronal type-specific mechanism. In addition to the NTS–PVN ascending circuits, the PVN–NTS descending circuits also play an important role in the regulation of BP. For example, optogenetic stimulation of corticotropin-releasing hormone-containing PVN neurons projecting to the NTS increases BP and HR [39]. Together, the present results, combined with previous findings, extend our understanding of the circuit-specific regulation of BP.

The PBN is a recipient of predominantly excitatory inputs from the NTS. Recent evidence points to an interaction between NTS and PBN neurons, including PBN-projecting noradrenergic NTS neurons mediating anorexia [12], and PBN-projecting CCK-expressing NTS neurons controlling feeding [40]. However, less is known about the role of the NTS–PBN circuit in the regulation of BP. The present results confirm that activation of NTS^PNMT^ neurons projecting to the LPBN significantly decreases BP and HR, amplifying our understanding of the contribution of the NTS–LPBN circuit to cardiovascular homeostasis.

Synaptic connections between the NTS and CVLM for cardiovascular regulation have been proposed [41]. Generally speaking, the depressor mechanisms in the NTS entail at least the following sequential steps: activation of glutamatergic NTS neurons, GABAergic neurons in the CVLM, and C1 neurons in the RVLM. In addition to glutamatergic NTS neurons projecting to the CVLM, the present findings enrich the concept that PNMT-containing neurons also project to the CVLM, although the neurochemical phenotypes of downstream neurons are undetermined. Moreover, NTS^PNMT^ neurons projecting to the CVLM are also responsible for the depressor response. It is noted that, unlike glutamatergic neurons that mediate a baroreflex-like effect, stimulation of CVLM-projecting NTS^PNMT^ neurons did not significantly affect HR. We suggest that this differential effect is attributable to different visceral inputs. Another interesting question is that, in spite of the principle of neurotransmitter coexistence [42], it is unknown whether NTS^PNMT^ neurons projecting to the CVLM contain glutamate. Taken together, we demonstrate that activation of NTS^PNMT^ neurons projecting to the PVN, LPBN, and CVLM induces depressor responses, morphologically and functionally revealing the circuit-specific control of cardiovascular homeostasis.

### Circuits Controlling the Pressor Response by NTS^PNMT^ Neurons

The NTS and RVLM are the main centers of the medullary circuits involved in cardiovascular control. The RVLM is the most important premotor sympathetic nucleus and is responsible for the generation and maintenance of sympathetic vasomotor tone [2]. In addition to receiving inhibitory inputs from the CVLM, the RVLM also receives excitatory projections from the brainstem, hypothalamus, limbic system, and cortex [43–45]. In particular, the NTS sends monosynaptic connections to the RVLM [46–49]. Moreover, NTS neurons projecting to the RVLM activate postsynaptic neurons *via* an excitatory neurotransmitter such as glutamate [50, 51]. However, the chemical phenotypes of such neurotransmitter candidates need to be fully determined. We assume that RVLM-projecting NTS^PNMT^ neurons most likely use adrenaline as the neurotransmitter but provide no evidence that axons of RVLM-projecting NTS^PNMT^ neurons release either adrenaline or glutamate or both for excitatory postsynaptic transmission. Promisingly, a genetically-encoded fluorescent sensor has been constructed that enables rapid and specific detection of neurotransmitters such as norepinephrine [52]. These genetic sensors are expected to reveal the neurotransmitter phenotypes of NTS^PNMT^ neurons.

In the present study, we stimulated not only the axons of NTS^PNMT^ neurons within the RVLM region but also the somata of retrogradely-labeled NTS^PNMT^ neurons from the RVLM. Both stimulation methods elicited a uniform pressor response; moreover, this robust pressor response was obtained in both anesthetized and conscious mice. Meanwhile, note that due to the adjacent anatomical positions of the RVLM and CVLM, the present findings could not exclude the possibility that optogenetic stimulation of axons of NTS^PNMT^ neurons located in either the CVLM or RVLM simultaneously activated those located in the other region; considering the working area and distance of the laser, and the position of the optical fiber, the illumination effect should be mainly ascribed to either nucleus. NTS^PNMT^ neurons projecting to the RVLM are expected to target C1 and non-C1 pre-sympathetic neurons to enhance sympathetic outflow. To unveil the physiological and clinical significance of this pressor response circuit, it is essential to determine the upstream monosynaptic inputs to the RVLM-projecting NTS^PNMT^ neurons, most likely from baroreceptors, chemoreceptors, nociception, and the hypothalamus. For example, the excitatory circuits from the NTS to the RVLM convey signals from the hypoxic activation of peripheral chemoreceptors [53–55], but it remains unknown whether NTS^PNMT^ neurons projecting to the RVLM contribute to such effects. This new circuit-specific pressor mechanism may be involved in the etiology of neurogenic hypertension.

### NTS^PNMT^ Neurons Contribute to Regulation of the Baroreflex

The arterial baroreflex plays a critical role in BP homeostasis, and re-setting of the baroreflex has been implicated in the onset and development of neurogenic hypertension [2, 3]. Barosensitive neurons of the NTS have been found to be phenotypically complex, some projecting to the CVLM and others projecting directly to the RVLM. In addition, glutamatergic but not GABAergic NTS neurons are considered to be barosensitive [1, 56]. So far, the neurochemical phenotypes and specific markers of barosensitive NTS neurons remain incompletely determined.

Chronic loss of catecholaminergic NTS neurons by anti-DβH conjugated to saporin results in lability of BP and impairment of baroreflex function [57]. However, none of these cardiovascular changes occur when TH/DβH-containing neurons are eliminated by 6-hydroxydopamine [58]. The opposing cardiovascular effects might be due to the selectivity of toxins for target neurons. To address these variable effects, the present results revealed that proportional ablation of NTS^PNMT^ neurons also impaired both bradycardic and tachycardic gain, confirming the important role of adrenergic NTS neurons in maintaining BP.

The pathway by which baroreceptor information reaches the RVLM remains undetermined. Although it is generally accepted that baroreceptor information accesses the RVLM principally *via* interneurons in a functionally-defined “depressor region” of the CVLM, a direct NTS–RVLM projection might also be taken into account. In the present loss-of-function experiments, after ablation of NTS^PNMT^ neurons projecting to the RVLM, the PE-evoked bradycardic response was significantly diminished, with no significant tachycardiac response. This finding was also consolidated by the present gain-of-experiments showing that activation of NTS^PNMT^ neurons projecting to the RVLM generated a pressor response and bradycardia. Thus, we propose that NTS^PNMT^ neurons, including those projecting to the RVLM, to some extent contribute to the regulation of the baroreflex. Nevertheless, we have not tested whether NTS^PNMT^ neurons projecting to the PVN, LPBN, and CVLM contribute to the regulation of the baroreflex. Since glutamatergic NTS neurons projecting to the CVLM are considered to mediate the baroreflex, such a role of NTS^PNMT^ neurons projecting to the CVLM should also be addressed. Therefore, it appears that NTS^PNMT^ neurons affect the baroreflex in a circuit-specific way.

## Summary

**Fig. 9:**
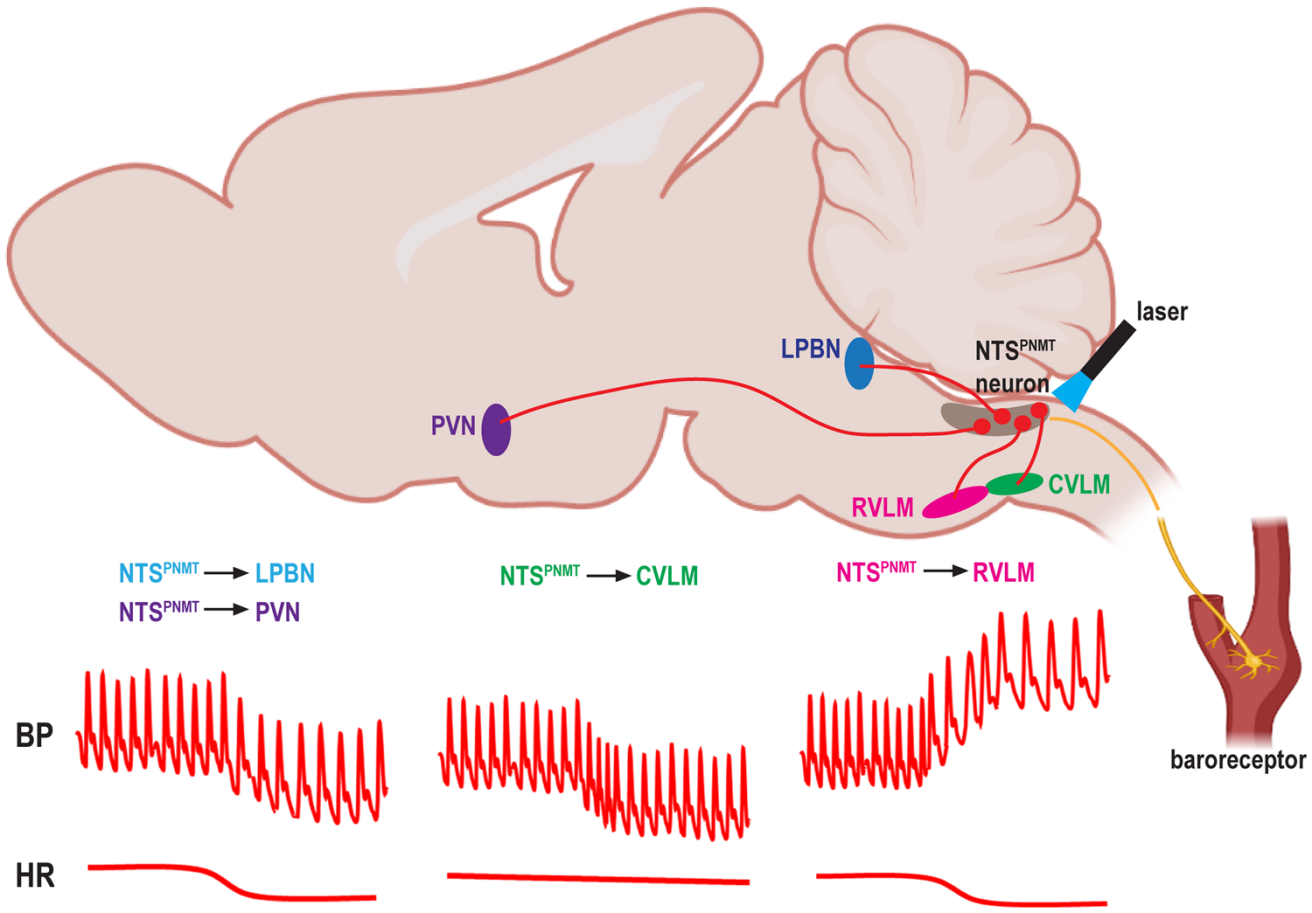
Summary of circuit mechanisms underlying the differential regulation of BP by NTS^PNMT^ neurons. Photostimulation of NTS^PNMT^ neurons projecting to the PVN, LPBN, and CVLM markedly reduces BP, whereas activation of those projecting to the RVLM induces a robust increase in BP and a decrease in HR.

In conclusion, our findings demonstrate that photostimulation of NTS^PNMT^ neurons projecting to the PVN, LPBN, and CVLM markedly reduces BP, whereas activation of NTS^PNMT^ neurons projecting to the RVLM induces a robust increase in BP and decrease in HR, suggesting neuronal phenotype- and circuit-specific mechanisms underlying homeostatic regulation of BP. Figure 9 is a schematic summary showing the circuit mechanisms underlying the differential regulation of BP by NTS^PNMT^ neurons. Moreover, NTS^PNMT^ neurons and those projecting to the RVLM contribute to the regulation of the baroreflex. These findings comprehensively enrich our understanding of how neuroanatomically- and functionally-defined neural circuits in the NTS spatiotemporally control cardiovascular activity.
